# Supplementary figures and images for: Integrated population modelling reveals potential drivers of demography from partially aligned data: a case study of snowy plover declines under human stressors
Source: PeerJ. 2021 Nov 15;9:e12475. doi: 10.7717/peerj.12475 (PMC8601057; doi:10.7717/peerj.12475)

# Texas

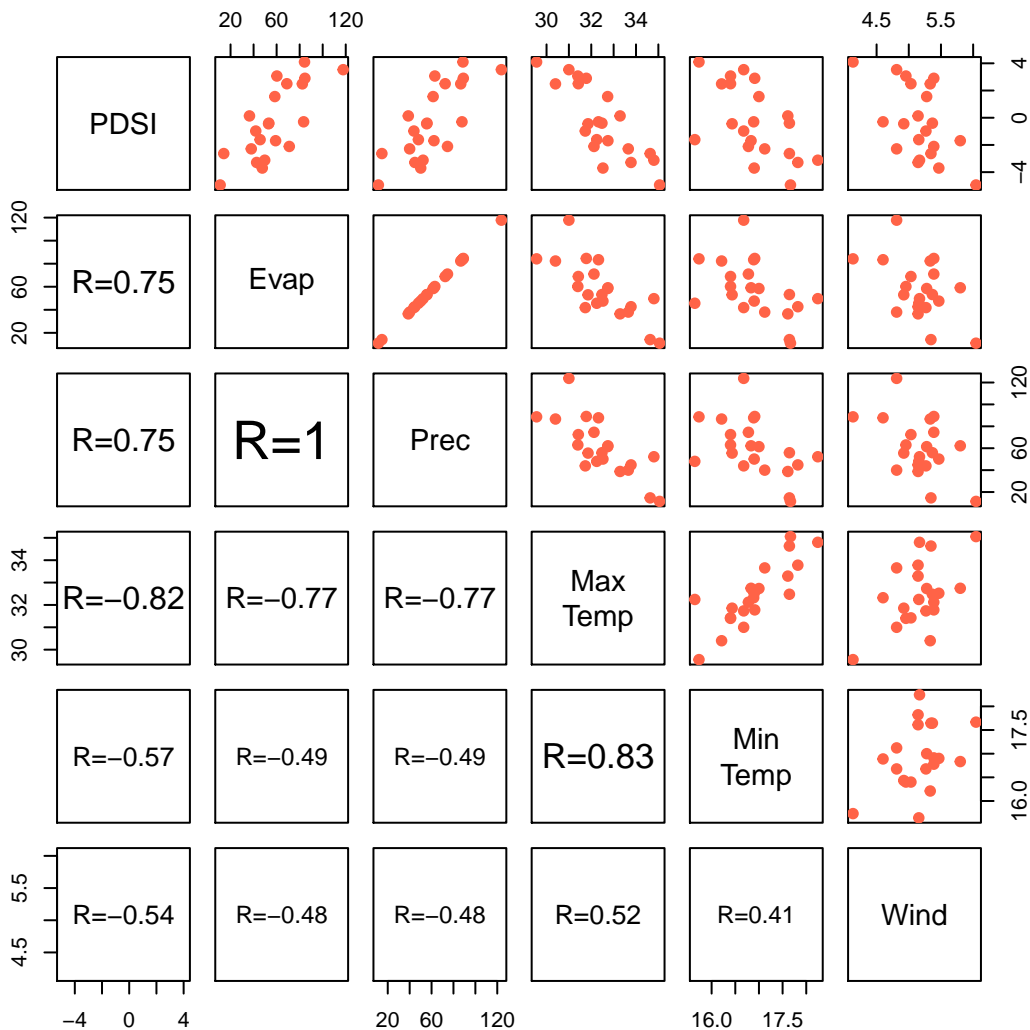

# New Mexico

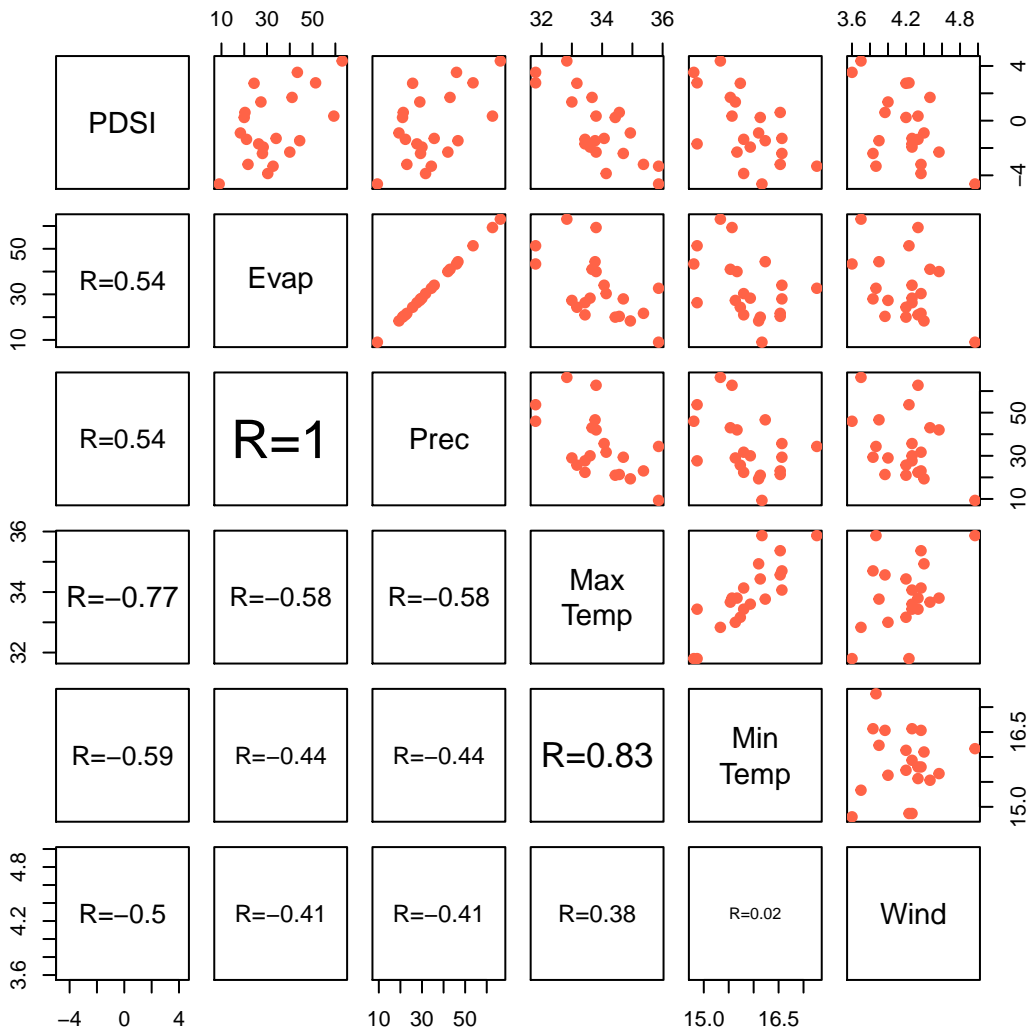

# Oklahoma

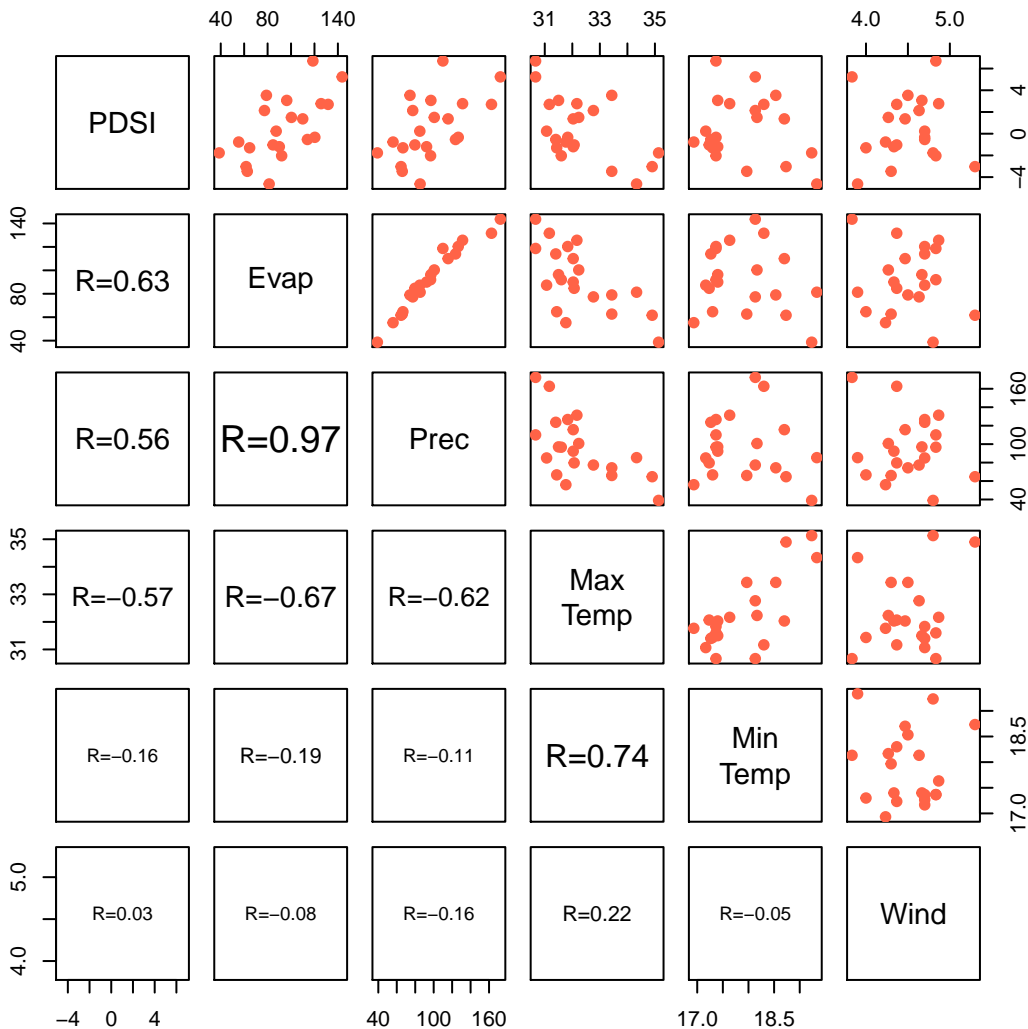

Supplement: Supplemental Information 12 — Scatter plots (upper triangle) and correlation coefficients (lower triangle) between each pair of the six environmental covariates that we originally considered, including Palmer drought severity index (PDSI), actual evapotranspiration (evap), precipitation (prec), maximum temperature (max temp), minimum temperature (min temp), and wind speed (wind). [file peerj-09-12475-s012.pdf]

Texas

New Mexico

Oklahoma

PDSI

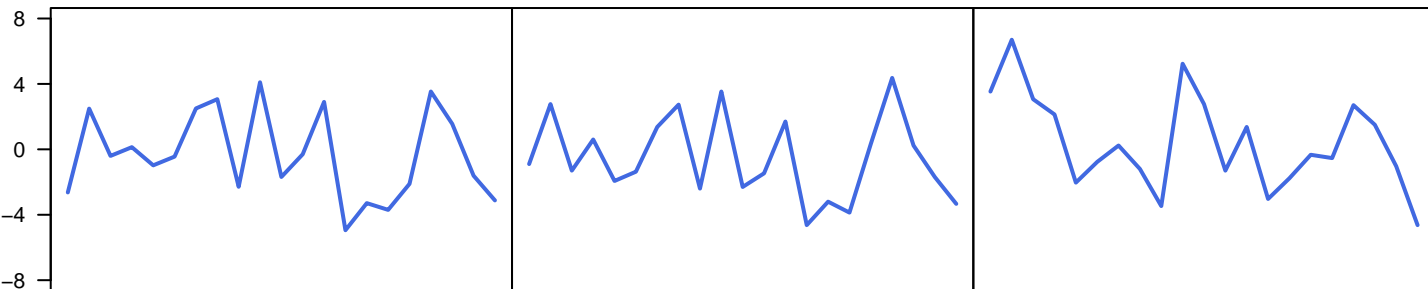

Min Temp

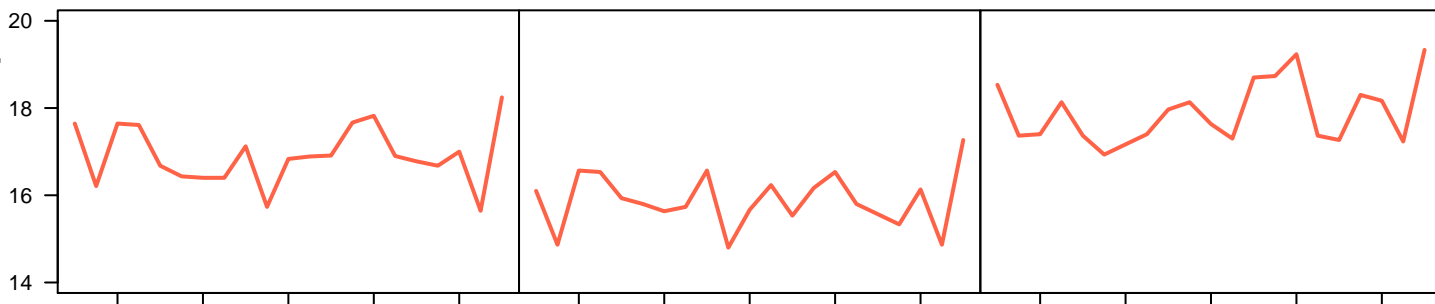

Wind

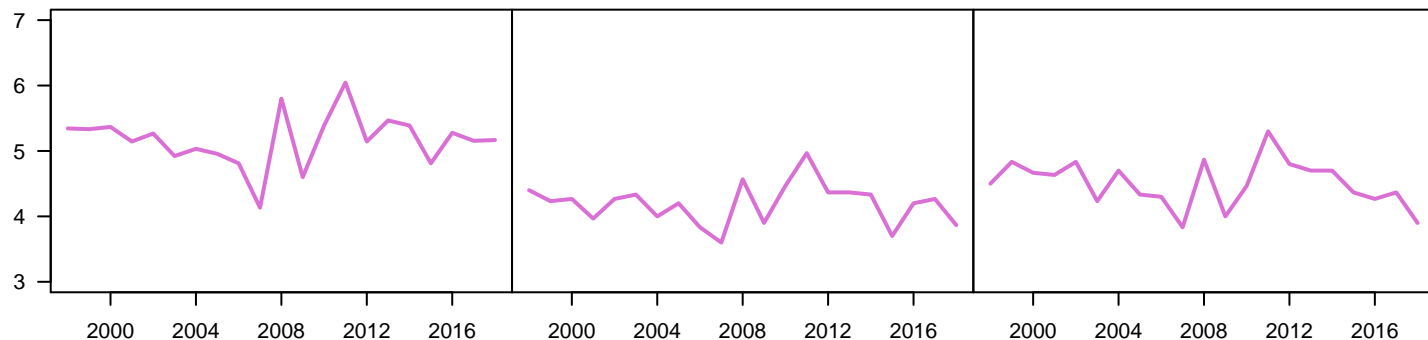

Year

Supplement: Supplemental Information 13 — Time series of Palmer drought severity index (PDSI), minimum temperature (min temp), and wind speed (wind) in Texas, New Mexico, and Oklahoma for the study period. [file peerj-09-12475-s013.pdf]
