# Supplementary material for: Integrated population modelling reveals potential drivers of demography from partially aligned data: a case study of snowy plover declines under human stressors
Source: PeerJ. 2021 Nov 15;9:e12475. doi: 10.7717/peerj.12475 (PMC8601057; doi:10.7717/peerj.12475)
Supplement: Supplemental Information 14 — Violin plots showing the region-specific effects of Palmer drought severity index (PDSI), minimum temperature (min temp), and wind speed (wind) on the probability of a clutch size of 2 or 3 and clutch fate in Texas (TX), New Mexico (NM), and Oklahoma (OK). The violin plot shows the entire posterior distribution, while the embedded boxplot shows the median (white dot), 50% Credible Interval (thick line), and 95% Credible Interval (thin line). [file peerj-09-12475-s014.pdf]

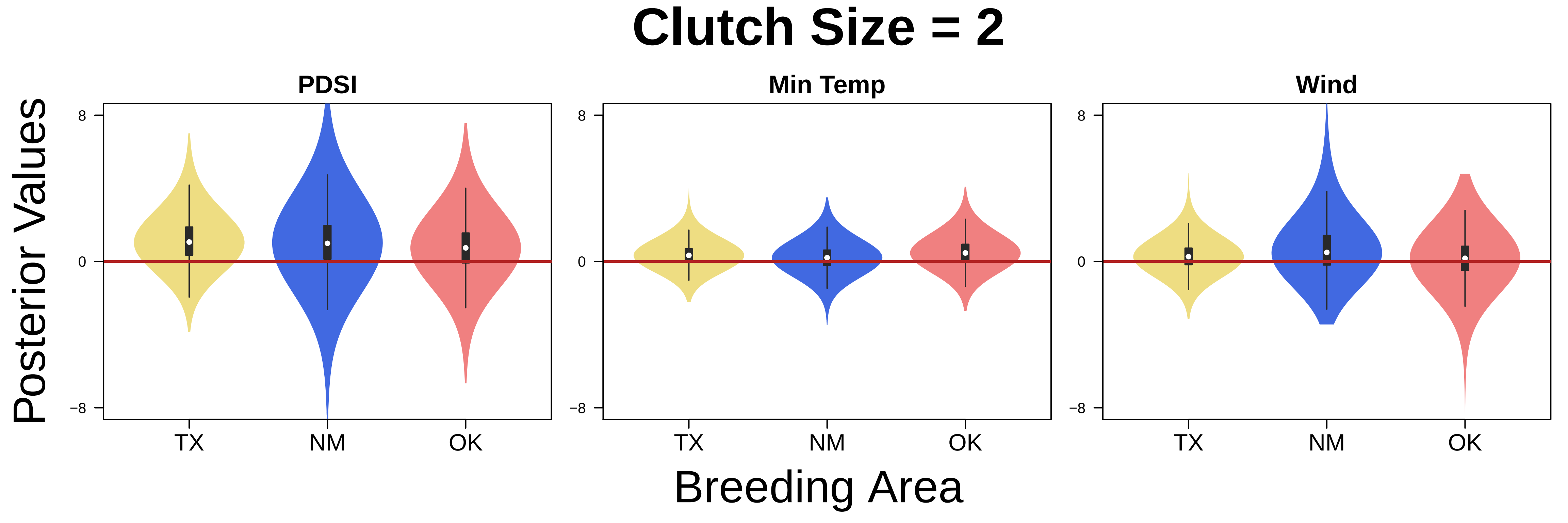

Clutch Size = 2
PDSI
Min Temp
Wind
Posterior Values
8
0
−8
TX
NM
OK
Breeding Area

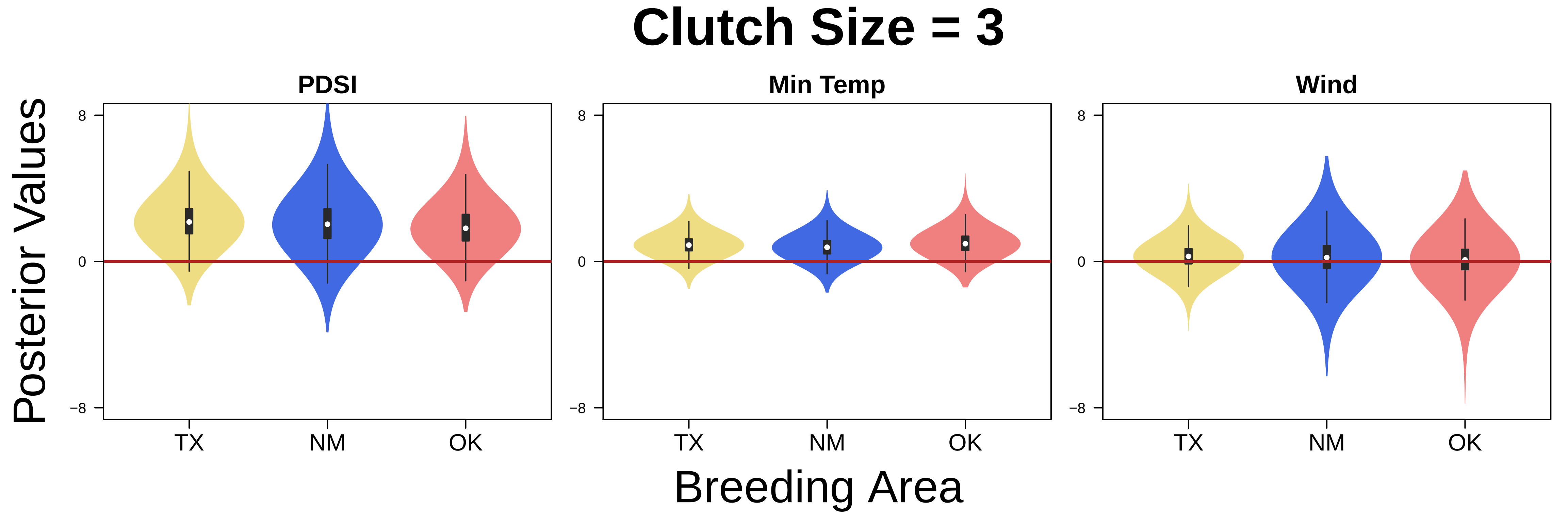

Clutch Size = 3
PDSI
Min Temp
Wind
Posterior Values
8
0
−8
TX
NM
OK
Breeding Area

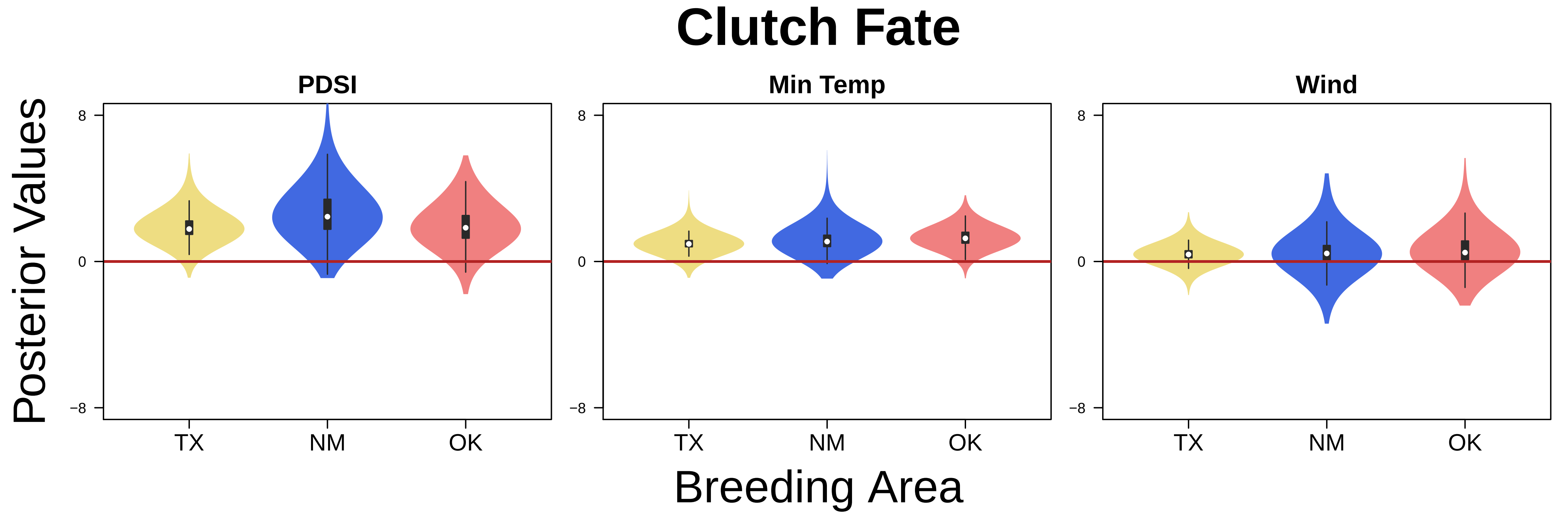
Clutch Fate
PDSI
Min Temp
Wind
Posterior Values
8
0
−8
TX
NM
OK
Breeding Area
