# Supplementary material for: Integrated population modelling reveals potential drivers of demography from partially aligned data: a case study of snowy plover declines under human stressors
Source: PeerJ. 2021 Nov 15;9:e12475. doi: 10.7717/peerj.12475 (PMC8601057; doi:10.7717/peerj.12475)
Supplement: Supplemental Information 15 — Violin plots showing the region-specific effects of Palmer drought severity index (PDSI), minimum temperature (min temp), and wind speed (wind) on the apparent survival of adult female, adult male, juvenile female, and juvenile male in Texas (TX), New Mexico (NM), and Oklahoma (OK). The violin plot shows the entire posterior distribution, while the embedded boxplot shows the median (white dot), 50% Credible Interval (thick line), and 95% Credible Interval (thin line). [file peerj-09-12475-s015.pdf]

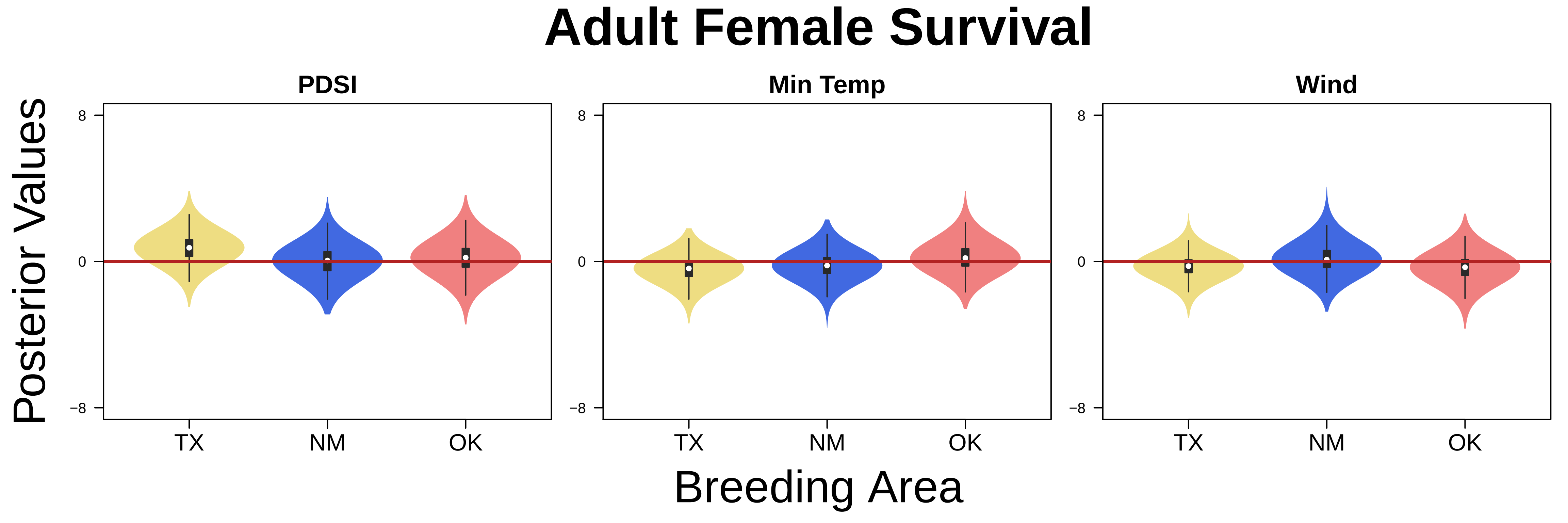

Adult Female Survival
PDSI
Min Temp
Wind
Posterior Values
8
0
−8
TX
NM
OK
Breeding Area

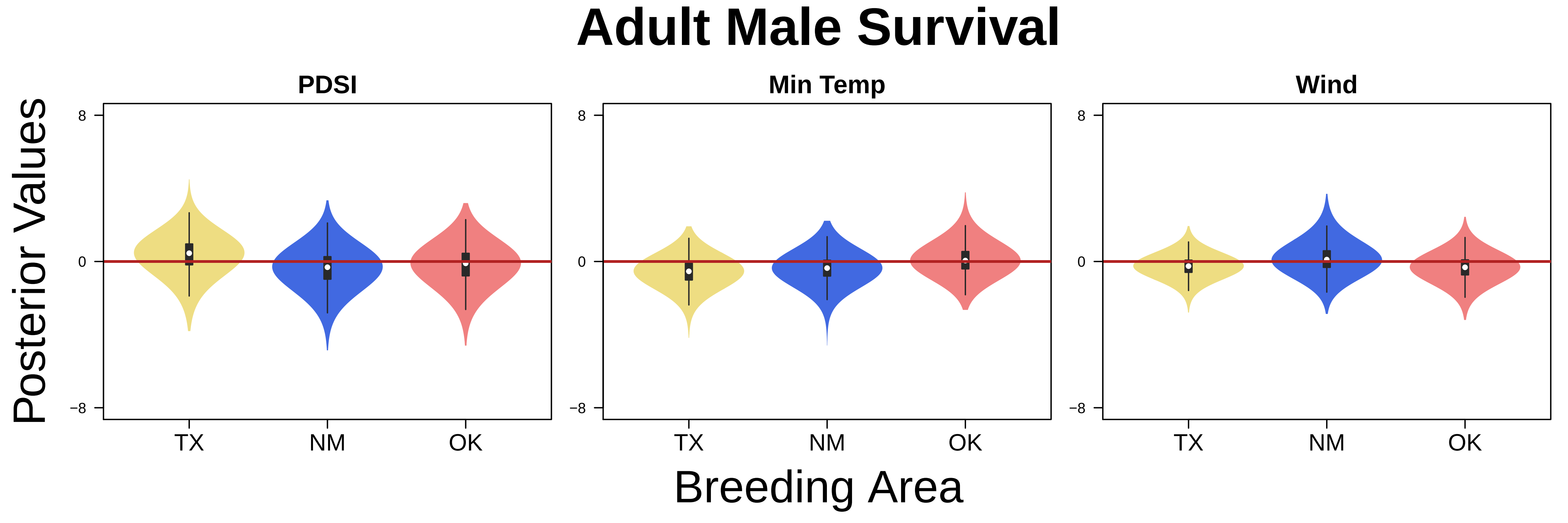

Adult Male Survival
PDSI
Min Temp
Wind
Posterior Values
8
0
−8
TX
NM
OK
Breeding Area

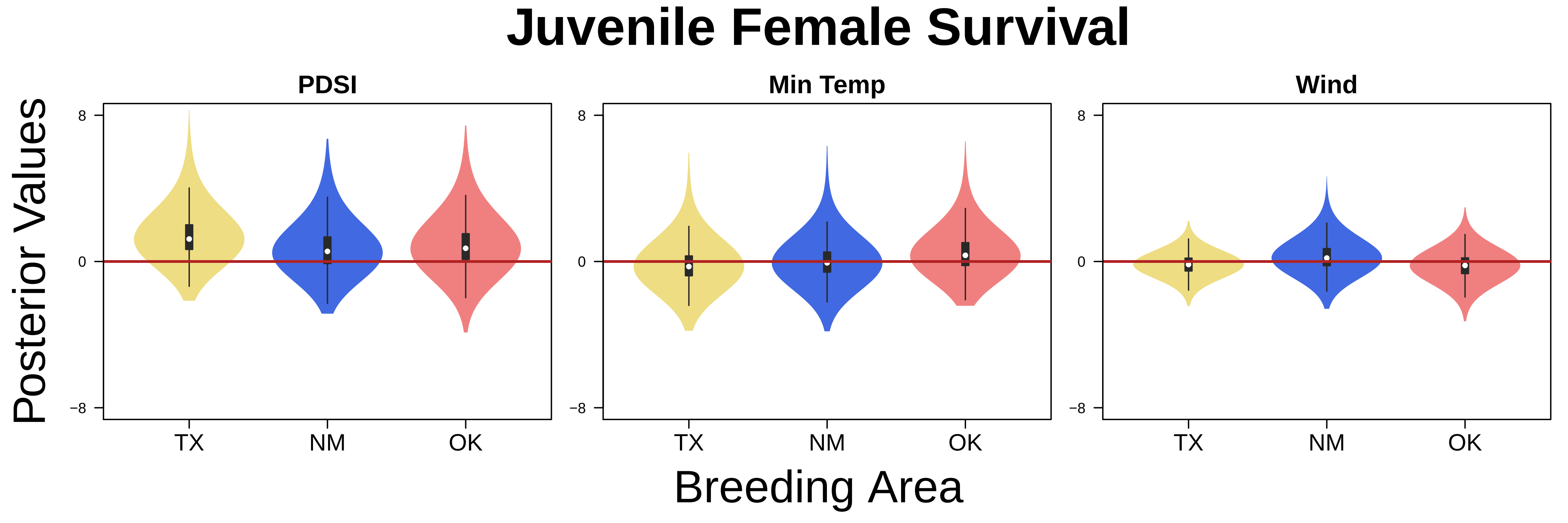
Juvenile Female Survival
PDSI
Min Temp
Wind
Posterior Values
8
0
−8
TX
NM
OK
Breeding Area

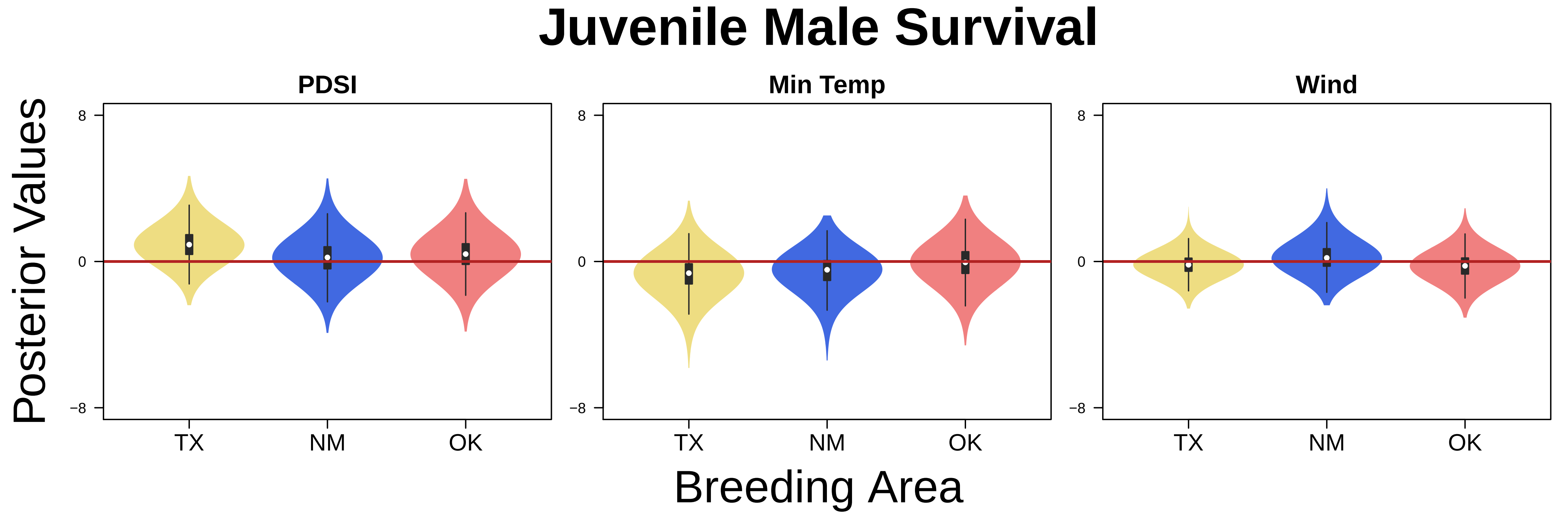

Juvenile Male Survival
PDSI
Min Temp
Wind
Posterior Values
8
0
−8
TX
NM
OK
Breeding Area
